# The Use of Raw Meat

**Published:** 1871-07

**Authors:** 


*The Use of Raw Meat* in the treatment of disease is objected to by Dr. M. R. Levi (*Girondale Veneto*) unless the said meat be other than that of the ox or the pig. He recommends the use of chicken, pigeon, or turkey meat for this purpose, as the others are apt to produce the tænia.

